# Factors associated with loss to follow-up in outpatient parenteral antimicrobial therapy: A retrospective cohort study

**DOI:** 10.1017/ice.2023.216

**Published:** 2024-03

**Authors:** Christina M. Kaul, Matthew Haller, Jenny Yang, Sadie Solomon, Maria R. Khan, Robert A. Pitts, Michael S. Phillips

**Affiliations:** 1 Division of Infectious Diseases, NYU Grossman School of Medicine, New York, New York; 2 Department of Population Health, NYU Grossman School of Medicine, New York, New York; 3 NYU Grossman School of Medicine, New York, New York; 4 Department of Medicine, NYU Grossman School of Medicine, New York, New York; 5 Department of Hospital Epidemiology, NYU Langone Health, New York, New York

## Abstract

We assessed factors associated with increased risk to loss of follow-up with infectious diseases staff in OPAT patients. Discharge to subacute healthcare facilities is strongly associated with loss to follow-up. We did not identify sociodemographic disparities. Poor communication between OPAT providers and subacute healthcare facilities remains a serious issue.

Outpatient parenteral antimicrobial therapy (OPAT) is utilized to treat infectious conditions in the outpatient setting.^1,2^ Due to the healthcare-wide shift of patient care to the outpatient setting, the use of OPAT has increased.^3,4^ With this increased reliance, processes, bundles, and outpatient programs have been developed, most of which focus on outcomes, including completion of therapy and prevention of readmission.^5–7^

Follow-up with infectious diseases (ID) staff in the outpatient setting can reduce hospital readmission.^8,9^ Our recent work reinforced that loss to follow-up leads to worsened outcomes, including increased risk of readmission.^7^ However, factors associated with loss to follow-up in the outpatient setting are not well studied, including sociodemographic factors. We sought to understand factors associated with increased risk of loss to follow-up with ID staff in OPAT patients.

## Methods

### Study design and setting

This retrospective cohort study was conducted at 4 hospitals within NYU Langone Health (NYULH) in the New York City region. The study was approved and granted a waiver of authorization and informed consent by the NYU Institutional Review Board.

At NYULH, a dedicated OPAT outpatient service is responsible for longitudinal follow-up until the antimicrobial course is completed. Not all patients were recommended for ID follow-up, per clinician judgment. The OPAT team includes physicians and three nurse practitioners. Patients were typically recommended for follow-up with an ID physician within 2–4 weeks of discharge, and weekly documented telephone or clinic visits with the ID nurse practitioner (NP). The number of attempts to reach the patient was recorded.

### Inclusion and exclusion criteria

All OPAT episodes for patients aged ≥18 years initiated with ID consultation during admission from January 1, 2017, to December 31, 2020, were screened. According to the Infectious Diseases Society of America clinical practice guideline, an OPAT episode was defined as the administration of at least 2 doses of an intravenous antimicrobial agent on different days without intervening hospitalization.^2^

### Data sources and extraction

Data sources included the electronic health record (EHR; Epic Systems, Verona, WI) and the NYULH OPAT registry. All patients discharged with a peripherally inserted central catheter (PICC) or midline catheter were screened by investigators for inclusion. The IRB-approved study personnel conducted comprehensive chart review of qualifying episodes.

### Data collection

Demographic factors assessed included sex assigned at birth, race, ethnicity, primary language, and the use of federal health insurance. Location of discharge was recorded: home, subacute rehabilitation center (SAR), hospital-based acute rehabilitation center (AR), or long-term care facility (LTCF). Race and ethnicity data were collected from the EMR, with standard of care to collect self-designated demographics. ZIP-code–level social vulnerability was classified according to the equitable distribution index (EDI) developed by the Centers for Disease Control and Prevention.^10^ The presence of medical comorbidities (eg, diabetes mellitus, chronic kidney disease, liver disease, malignancy, and HIV), follow-up duration, and hospital of admission were recorded. Loss to follow-up with ID staff was defined as the absence of an encounter related to the OPAT course after discharge. In-person or virtual encounters were considered as seen.

### Statistical analysis

Data were analyzed using Stata version 18 software (Statacorp, College Station, TX) and Excel 2019 software (Microsoft, Redmond, WA). Descriptive statistics were used. Multivariate logistic regression was used to examine factors associated with loss to follow-up, adjusted for key covariates including chronic medical condition, hospital of admission, and line duration. A sensitivity analysis was run, removing race and ethnicity from the model. *P* ≤ .05 was considered statistically significant.

## Results

In total, 5,951 cases of adult patients discharged with vascular access were identified. 1,846 instances were determined to be qualifying OPAT courses.

Overall, 1,528 patients (82.7%) were recommended for follow-up with ID staff in the outpatient setting. Of this subset, 40.5% were female. Furthermore, 56.2% were White, 15.0% were Hispanic, 13.0% were Black,7.4% were Asian, and 5.8% were other race. Also, 52.4% had midline catheters and 47.6% had PICCs. The insurance of 26.8% of patients was commercial, 16.7% was Medicaid, and 56.2% was Medicare.

In total, 1,110 patients (72.6%) were seen by ID staff at least once in the outpatient setting. Furthermore, 418 patients (27.4%) were not seen. Of patients seen, 981 (88.4%) were seen by the ID physician and 129 (11.6%) were only seen by the ID nurse practitioner. Patients were seen or contacted by the ID physician a median of twice during their follow-up period. Patients were seen or contacted by the ID nurse practitioner a median of once during their follow-up period. The median follow-up period for patients not lost to follow-up was 21 days. Of patients lost to follow-up, 43.5% were female. 56.7% were White, 14.4% were Hispanic, 14.6% were Black, 7.2% were Asian, and 5.7% were other race. Also, 43.8% were discharged home, 43.8% were discharged to an SAR facility, 4.1% were discharged to an AR facility, and 6.9% were discharged to an LTCF. Further characteristics of the study population are listed in Table 1.

**Table 1.** Participant Population

Patient Characteristic (N=1,528)	No. (%)^a^
Age, median y (IQR)	65 (54–75)
Sex, female	619 (40.5%)
**Race and ethnicity**	
Non-Hispanic White	858 (56.2)
Hispanic	229 (15.0)
Black	199 (13.0)
Asian	113 (7.4)
Other	89 (5.8)
HIV	62 (4.1)
Intravenous drug use	39 (2.6)
**Insurance type**	
Private	410 (26.8)
Medicaid	255 (16.7)
Medicare	859 (56.2)
**Line type**	
PICC	727 (47.6)
Midline	801 (52.4)
**Discharge location**	
Home	1093 (59.2)
SAR	563 (30.5)
AR	125 (6.8)
LTCF	65 (3.5)
Follow-up period, mean d (IQR)	21 (10–36)
Encounters with ID physician, median (IQR)	2 (1–3)
Encounters with ID nurse practitioner, median (IQR)	1 (0–3)

Note. HIV, human immunodeficiency virus; PICC, peripherally inserted central catheter; SAR, subacute rehabilitation facility; AR, acute rehabilitation facility; LTCF, long-term care facility; ID, infectious diseases; IQR, interquartile range.

a Units unless otherwise specified.

In the model, we detected a significant association between loss to follow-up with ID staff and discharge to an SAR facility (OR, 3.24; 95% CI, 2.35–4.47; *P* < .001) or LTCF (OR, 5.91; 95% CI, 2.89–12.03; *P* < .001). A similar association was not seen for discharge to an AR facility. There was also an association between loss to follow-up and federal insurance (OR, 1.493; 95% CI, 1.04–2.14; *P* = .029). We did not identify disparities based on sex, race or ethnicity, primary language, or EDI (Table 2). In our sensitivity analysis, removing race and ethnicity from the model, EDI was not statistically significant (OR, 1.57; 95% CI, −0.96 to 2.59; *P* = .074).

**Table 2.** Patient Risk Factors in Loss to Follow-Up with Infectious Diseases

Variable	OR	95% CI	*P* Value
Sex, female	1.22	(0.95–1.56)	.123
**Ethnicity**			
Asian	0.88	(0.53–1.44)	.614
Black	0.97	(0.67–1.42)	.886
Hispanic	0.81	(0.56–1.43)	.255
Other	0.84	(0.49–1.43)	.514
Non-English speaking	1.12	(0.80–1.56)	.503
EDI	1.64	(0.97– 2.77)	.066
**Discharge location**			
SAR	3.13	(2.40–4.08)	**<.001**
AR	0.76	(0.42–1.39)	.378
LTC	6.38	(3.35–12.14)	**<.001**
Federal insurance	1.61	(1.17–2.21)	**.003**

Note. OR, odds ratio; CI, confidence interval; EDI, equitable distribution index; SAR, subacute rehabilitation facility; AR, acute rehabilitation facility; LTCF, long-term care facility. Bold indicates statistical significance.

In our study, >25% of patients recommended for outpatient follow-up with ID staff were not seen. We did not identify disparities in loss to follow-up based on sex, race, or ethnicity. Patients discharged to SAR facilities were at a significantly higher risk of being lost to follow-up than those discharged home. Reasons for this difference remains unclear, but they could be related to facility staffing issues or communication challenges with outpatient providers. Lack of follow-up certainly puts patients at risk for worse outcomes, including increased risk for readmission and catheter-related bloodstream infections.^7,11^ Discharge to an AR facility was not associated with an increased loss to follow-up, which is not unexpected given that it is hospital-affiliated with a shared EHR. Loss to follow-up also leads to underutilization of dedicated resources, put in place to optimize treatment completion and patient safety. This finding is not surprising given difficulty in communication that tends to exist between outpatient healthcare providers and these facilities. We also noted increased loss to follow-up among patients utilizing federal insurance. We did not detect reasons for this interesting, but this trend warrants further investigation given the large proportion of patients with federal insurance nationwide. We did note a nonsignificant increased risk of loss to follow-up with EDI, raising the possibility of a β (beta) error. As such, we cannot draw conclusions based on the significance of EDI, and further research should be pursued to address the role of EDI in ID loss to follow-up.

Our study had several strengths, including large sample size, long-term data, and multiple hospitals across a wide catchment area. Our study also had several limitations. The study was conducted in a single health system, possibly limiting generalizability. Our data lacked EMR integration with subacute centers. The study population was predominantly insured, potentially obscuring social factors known to contribute to loss to follow-up. More data are needed among diverse patient populations to understand barriers to successful OPAT delivery and care.

Discharge to an SAR or LTC facility was strongly associated with loss to follow-up with ID staff. These findings further underscore the importance of maintaining links to patients discharged to subacute healthcare facilities and that interventions geared toward these populations should be utilized. Potential next steps include qualitative studies on patient and key stakeholder experiences. Transitions of care remain a weakness in healthcare delivery and must be improved upon in OPAT.
